# Child-to-Adult Liver Transplantation With Donation After Cardiac Death Donors: Three Case Reports

**DOI:** 10.1097/MD.0000000000002834

**Published:** 2016-02-18

**Authors:** Liangshuo Hu, Xuemin Liu, Xiaogang Zhang, Liang Yu, Huanchen Sha, Ying Zhou, Min Tian, Jianhua Shi, Wanli Wang, Chang Liu, Kun Guo, Yi Lv, Bo Wang

**Affiliations:** From the Department of Hepatobiliary Surgery, First Affiliated Hospital of Xi’an Jiaotong University, Xi’an, Shaanxi, China.

## Abstract

Development of organ transplantation is restricted by the discrepancy between the lack of donors and increasing number of patients. The outcome of pediatric donors transplanted into adult recipients especially with donation after circulatory death (DCD) pattern has not been well studied. The aim of this paper is to describe our experience of 3 successful DCD donor child-to-adult liver transplantations lately.

Three DCD donors were separately 7, 5, and 8 years old. The ratio between donor graft weight and recipient body weight was 1.42%, 1.00%, and 1.33%, respectively. Ratio between the volume of donor liver and the expected liver volume was 0.65, 0.46, and 0.60. Splenectomy was undertaken for the second recipient according to the portal vein pressure (PVP) which was observed during the operation.

Two out of 3 of the recipients suffered with acute kidney injury and got recovered after renal replacement therapy. The first recipient also went through early allograft dysfunction and upper gastrointestinal bleeding. The hospital course of the third recipient was uneventful. After 1 year of follow-up visit, the first and second recipients maintain good quality of life and liver function. The third patient was followed up for 5 months until now and recovered well.

DCD child-to-adult liver transplantation should only be used for comparatively matched donor and recipient. PVP should be monitored during the operation. The short-term efficacy is good, but long-term follow-up and clinical study with large sample evaluation are still needed.

## INTRODUCTION

Development of organ transplantation is restricted by the discrepancy between the lack of donors and increasing number of patients. Since 1994, the World Health Organization has encouraged organ donation after circulatory death (DCD) worldwide.^1^ DCD donors appropriately mitigate the serious situation of organ shortage, but still could not meet the large demands. The application and efficacy of marginal donor liver as well as reduced-size graft despite significant size mismatch become important topics for liver transplantation.

The outcome of pediatric donors transplanted into adult recipients especially with DCD pattern has not been well studied. Weight of pediatric donor liver is considerably less than the standard liver weight of adult patient, the difference comes with excessive portal venous inflow and portal hypertension which could lead to small-for-size syndrome (SFSS).^2^ And as DCD grafts, ineluctable warm ischemia time could cause more postoperative complications such as acute kidney injury (AKI),^3^ early allograft dysfunction (EAD),^4^ and more hepatobiliary complications.^5^ It is a big challenge for the surgeons to choose using such graft rather than discarded it. Here we describe the experience of three successful DCD donor child-to-adult liver transplantations in our center lately.

## PATIENTS AND METHODS

### DCD Donor Information

The DCD donor grafts were from 3 boys (Table 1). They all suffered with irreversible brain injury which was confirmed with the Maastricht categories of DCD type III.^6^ The donor grafts were harvested via double in situ perfusion with combined liver and kidney rapid resection technique.

**TABLE 1:**
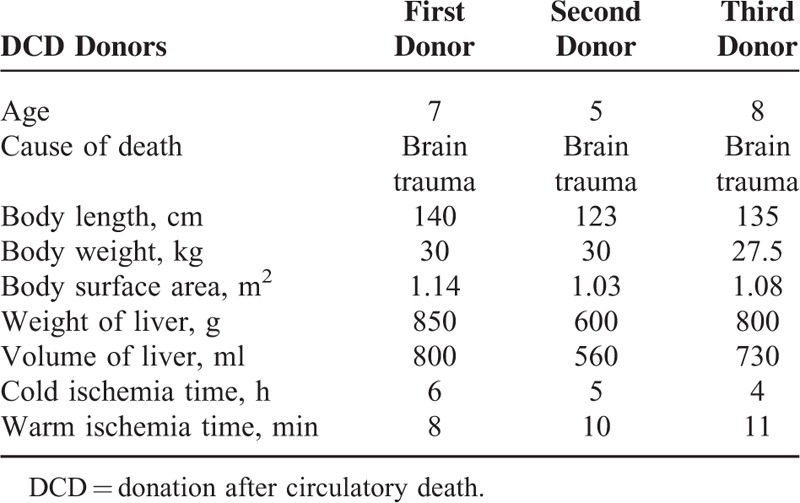
Donor Information

The first donor is 7 years old. The body length (BL) and body weight (BW) were 140 cm and 30 kg with a 1.14 m^2^ body surface area (BSA), respectively. The weight and volume of the first donor liver were separately 850 g and 800 ml. The warm ischemia time (time from arrest to cold flush or regional perfusion) was 8 minutes and the cold ischemia time of them was 6 hours.

The second donor is 5 years old. His BL and BW were 123 cm and 30 kg (BSA 1.03 m^2^). The donor liver weight 600 g and its volume was 560 ml. The warm and cold ischemia time were 10 minutes and 5 hours, respectively.

The third donor is 8 years old. He was 135 cm tall and weighed 27.5 kg (BSA 1.08 m^2^). The weight and volume of liver were separately 800 g and 730 ml, respectively. The warm ischemia time was 11 minutes and the cold ischemia time of them was 4 hours.

### Recipient Information

Three adult recipients were well explained with all the details of the potential risks and surgical complications (Table 2). Operations were performed with the informed decision of the patients and their family and the approval of the hospital ethics committee.

**TABLE 2:**
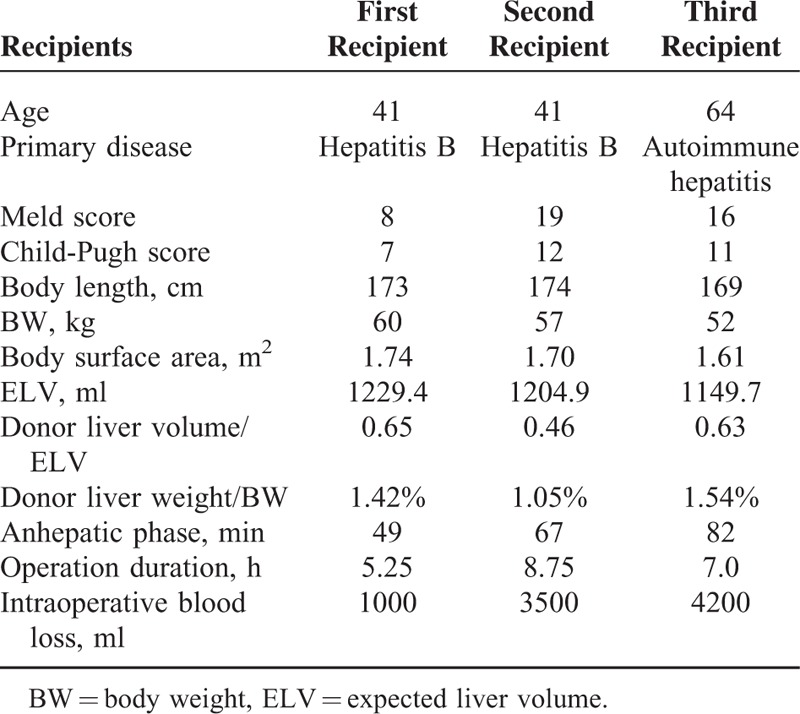
Recipient Information

All the patients accepted the orthotopic liver transplantation (OLT) and the second recipient received splenectomy at the same time. Hepatic artery anastomosis was performed between the common hepatic artery of donor and the bifurcation of gastroduodenal-proper hepatic artery with 7-0 Prolene suture. Duct-to-duct anastomosis was used to reconstruct the bile duct with a T-tube indwelled for biliary drainage. Portal vein pressure (PVP) was monitored as reported before.^7^ The first and second recipient received an immunosuppressive treatment with tacrolimus + mycophenolate mofetil (MMF) after surgical intervention and the third patient took cyclosporine (CsA) + MMF. ALL of them underwent anticoagulation treatment using low-molecular-weight heparins followed by warfarin.

The first recipient is a 41-years-old male. His primary disease was hepatitis B cirrhosis. He once underwent upper gastrointestinal bleeding and splenectomy, and the model for end-stage liver disease (MELD) score and Child-Pugh score were separately 8 and 7. The BL and BW were 173 cm and 60 kg with a 1.74 m^2^ BSA, respectively. The expected liver volume (ELV) which is calculated with the formula (ELV = 613 × BSA + 162.8) was 1229.4 cm^3^.^8^ The ratio between the volume of donor liver and ELV was 0.65. The ratio between donor graft weight and recipient BW were 1.42%. The anhepatic phase of the first recipient lasted 49 minutes. The PVP before and early after transplantation were separately 32 and 28 cmH_2_O. The operation time was 5.25 hours and the amount of intraoperative bleeding was 1000 ml.

The second recipient is also a 41-years-old male and his primary disease was hepatitis B cirrhosis as well. With hepatic encephalopathy (HE, grade II), the MELD score and Child-Pugh score for the second recipient were 19 and 12, respectively. His BL and BW were 174 cm and 57 kg (BSA 1.70 m^2^). His ELV was 1204.9 cm^3^. The ratio between the volume of donor liver and ELV was 0.46. And the ratio between donor graft weight and recipient BW was 1.05%. The anhepatic phase of the second recipient was 67 minutes. The PVP before transplantation was 25 cmH_2_O (decreased from 35 cmH_2_O before splenectomy) and the early postoperative PVP was 20 cmH_2_O. The operation time was 8.75 hours and the amount of intraoperative bleeding was 3500 ml.

The third recipient is a 64-years-old female whose primary disease was autoimmune hepatitis-related cirrhosis. The third recipient got the MELD score and Child-Pugh score of 16 and 11, respectively. She was 169 cm tall and weighed 52 kg (BSA 1.61 m^2^). Her ELV was 1149.7 cm^3^. The ratio between the volume of donor liver and ELV was 0.63. And the ratio between donor graft weight and recipient BW was 1.54%. The anhepatic phase of the third recipient was 56 minutes. The PVP before transplantation was 20 cmH_2_O and the early postoperative PVP was 18 cmH_2_O. The operation time was 7 hours and the amount of intraoperative bleeding was 4200 ml.

### Outcomes and Follow-Up

**FIGURE 1:**
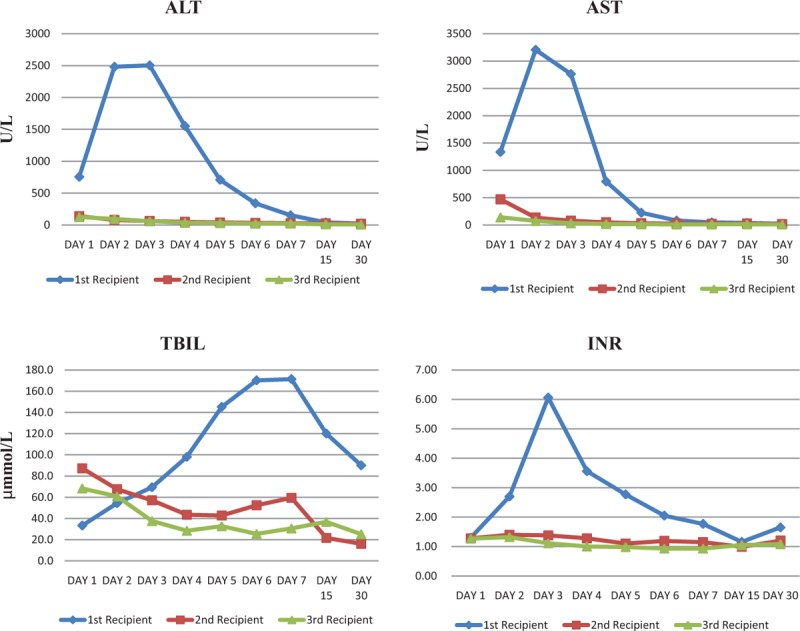
Liver function was tested after transplantation. The peak transaminase and total bilirubin (TBIL) for the first patient appeared on Days 2 and 7 post-OLT, respectively. The variation of International Normalized Ratio (INR) has the same trend with transaminase. The liver recovered with the help of plasma exchange therapy after Day 7 post-OLT. The liver function recovered well after transplantation for Patients 2 and 3.

**FIGURE 2:**
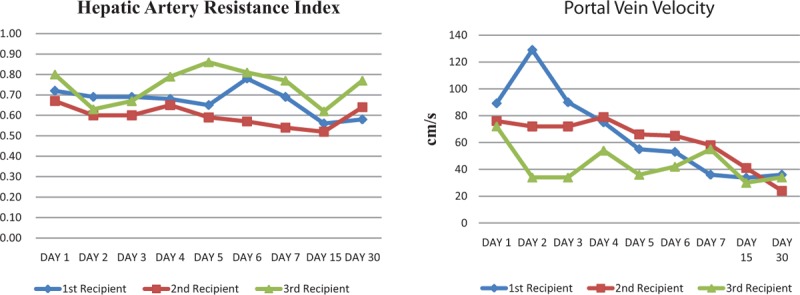
Unlike the other 2, the portal vein velocity for the first patient went through a rise and fall change which might lead to portal hyperperfusion and contribute to poor graft function after transplantation. No hepatic arterial vasoconstriction or hepatic artery thrombosis was found.

The post-OLT course was complicated by EAD after transplantation for the first recipient with no bile drainage through the T-tube, prolonged liver cholestasis, and the peak transaminase was over 2000 U/L for days. The peak blood ammonia was 141 μmol/L. The liver function was recovered after thrice plasma exchange after Day 7 (Figure 1). Resistance index of hepatic artery and portal vein velocity were monitored by abdominal ultrasound examination^2^ (Figure 2). He also suffered AKI and restored after continuous renal replacement therapy (CRRT), without notable water–sodium retention and electrolyte disorders. Pulmonary infection occurred on postoperative Day 7 and was controlled via antibiotic treatment. On Day 9, iliac vein thrombosis of double lower limbs was observed, and venacavography was performed with inferior vena cava filter (IVCF) placement. Twenty days following OLT, the patient unexpectedly returned to the intensive care unit for upper gastrointestinal bleeding and recovered after 2 days of conservative treatment. At 3 months post-OLT, tuberculosis was found and the patient was treated with combined anti-tuberculosis treatment (Rifampin 0.45 QD, Isoniazide 0.3 QN, Ethambutol 0.75 QD).

Temporary renal function damage also occurred in the second recipient on Days 1 to 3 and was treated with CRRT. At 1 month following OLT, cytomegalovirus (CMV) DNA test shown positive result and he received Valcyte treatment for a month till the test result turned negative.

The hospital course was uneventful for the third recipient.

After 1 year of follow-up visit, the first and second recipients maintain good quality of life and liver function. T-tube was removed 2 months post-OLT and no vascular or biliary complication was found. The third patient was followed up for 5 months until now and she recovered well. The case report was approved of the Ethical Committee of the First Affiliated Hospital of Xi’an Jiaotong University and approved of the patients consent.

## DISCUSSION

Decades after liver transplantation been widely studied, organ shortage remains the main problem which restricts the qualitative advancement of this treatment. To save more patients on the waiting list, surgeons usually have to take chances to use organs from the expanded donor pool. As the proportion of adult recipients is much larger than that of pediatric recipients, donors from the pediatric age group are becoming more likely to be directed to adult recipients. As a small-for-size liver for an adult, the most important potential risk is SFSS. It is reported by previous studies that grafts must be greater than 0.8% of the recipient BW or >40% of ELV to avoid postoperation SFSS.^9–11^ To reach such standard, we carefully choose recipients with low BW when allocated the liver. To our certain knowledge, the second boy in this paper is the youngest DCD donor whose liver is successfully transplanted to an adult. But even the 3 cases in this report are all well match with the standard mentioned above, EAD still occurred in the first recipient. We believe that plasma exchange and molecular adsorbent recirculating system could be useful in such situation and help the grafts to recover.^12,13^

Another reason which may cause the EAD for the first recipient is relatively high PVP after transplantation. Yagi et al found a poor outcome for the graft early postoperative PVP elevation to 20 mmHg.^14^ Zhou et al^7^ found the improvement in esophageal varices could be observed in the whole liver transplantation group 2 weeks post-OLT, whereas no change was observed in the living donor liver transplantation group. This may explain the upper gastrointestinal bleeding for the first recipient. The following report by Feng et al shown the advantage of splenectomy which could prevent the occurrence of SFSS by leading a decrease in the portal flow and PVP and a significant increase in the arterial flow.^15,16^ Nevertheless, there is also considerable increase of surgical risk and infection for splenectomy.^17,18^ On the other hand, recent studies suggest spleen plays an important role in regulating the immune system, metabolism, and endocrine function.^19^ But the main reason which increases the morbidity and mortality in asplenia or postsplenectomy states comes from immunological and infectious complications.^20^ We recommend intraoperative monitoring of PVP and splenectomy which is necessary only if the patient has portal hypertension to protect the donor graft and prevent postoperative gastrointestinal bleeding. Long-term risk of overwhelming post-splenectomy infections should be aware and immediate medical attention is needed in response to every febrile episode.^21^

The outcomes of the Child-to-Adult liver transplantation have not being well studied. But both Ruud and Emre research suggested a greater incidence of vascular complications and worse 1-year graft survival.^22,23^ They both considered the discrepancies in the size of the donor as compared with the recipient might be the main reason caused the bad prognosis. Size mismatch is not only the risk for hyper-perfusion injury but also causes blood stream slow at the first hepatic portal which could induce thrombosis. They suggested using donor-to-recipient BSA ratio to be the evaluation criteria before OLT. Based on above reasons, they both suggested anticoagulation therapy to the recipient to prevent vascular complications, especially hepatic artery thrombosis. We applied an anticoagulation protocol with short-term low molecular weight heparin right after transplantation followed by warfarin for about half a year and no vascular complication has been found yet. However, long-term following-up is still needed.

These 2 papers above found a relationship between ischemia time and vascular complications. Both cold and warm ischemia time have significant impact on the prognosis post-OLT. In this study, we first report DCD Child-to-Adult liver transplantation with a good short-term efficacy. Since March 2010 when the Chinese organ donation system been developed, the ratio of DCD liver transplantation have stepwise become dominate.^24^ The main challenge of current DCD practice is prolonged warm ischemia time which could lead to a series of post-OLT disorders, such as AKI and hepatobiliary complications.^25^ Two out of 3 of our patients suffered with AKI and needed CRRT treatment. Leithead et al^3^ reported that DCD liver transplantation is associated with an increased frequency of AKI. There is no evidence that AKI is related to pediatric or size mismatched grafts. CRRT could help renal function recovery, maintaining a stable blood volume and removing inflammation factors.^26^ Although usage of T-tube to prevent biliary complication remains controversial, it is recommended when the bile duct diameter is less than 7 mm.^27,28^ In the size mismatch situation of Child-to-Adult liver transplantation, T-tubes are used in the duct-to-duct anastomosis biliary tract reconstruction in all 3 cases of this study with good outcomes. We also believe that the ischemia time and donor graft injury could be minimized with rational intensive care unit management and lead to a good recovery for the recipient.^29^

## CONCLUSIONS

In conclusion, we suggest DCD pediatric donor liver could only be used for comparatively matched adult recipient. PVP should be monitored during the operation. The short-term efficacy is good, but long-term follow-up and clinical study with large sample evaluation are still needed.

## References

[1] Daemen JW Kootstra G Wijnen RM Nonheart-beating donors: the Maastricht experience. *Clin Transpl* 1994; 7:303–316. 7547551

[2] Yagi S Iida T Taniguchi K Impact of portal venous pressure on regeneration and graft damage after living-donor liver transplantation. *Liver Transpl* 2005; 11:68–75. 15690538 10.1002/lt.20317

[3] Leithead JA Tariciotti L Gunson B Donation after cardiac death liver transplant recipients have an increased frequency of acute kidney injury. *Am J Transplant* 2012; 12:965–975. 22226302 10.1111/j.1600-6143.2011.03894.x

[4] Lee DD Singh A Burns JM Early allograft dysfunction in liver transplantation with donation after cardiac death donors results in inferior survival. *Liver Transpl* 2014; 20:1447–1453. 25179581 10.1002/lt.23985

[5] Dubbeld J van Hoek B Ringers J Biliary complications after liver transplantation from donation after cardiac death donors: an analysis of risk factors and long-term outcome from a single center. *Ann Surg* 2015; 261:e64. 24401915 10.1097/SLA.0000000000000513

[6] Morrissey PE Monaco AP Donation after circulatory death: current practices, ongoing challenges, and potential improvements. *Transplantation* 2014; 97:258–264. 24492420 10.1097/01.TP.0000437178.48174.db

[7] Jiang SM Zhang QS Zhou GW Differences in portal hemodynamics between whole liver transplantation and living donor liver transplantation. *Liver Transpl* 2010; 16:1236–1241. 21031538 10.1002/lt.22138

[8] Li FG Yan LN Li B [Estimation formula of standard liver volume for Chinese adults]. *Sichuan Da Due Xue Bao Yi Xue Ban* 2009; 40:302–306. 19462913

[9] Kiuchi T Kasahara M Uryuhara K Impact of graft size mismatching on graft prognosis in liver transplantation from living donors. *Transplantation* 1999; 67:321–327. 10075602 10.1097/00007890-199901270-00024

[10] Ben-Haim M Emre S Fishbein TM Critical graft size in adult-to-adult living donor liver transplantation: impact of the recipient's disease. *Liver Transpl* 2001; 7:948–953. 11699030 10.1053/jlts.2001.29033

[11] Inomata Y Kiuchi T Kim I Auxiliary partial orthotopic living donor liver transplantation as an aid for small-for-size grafts in larger recipients. *Transplantation* 1999; 67:1314–1319. 10360583 10.1097/00007890-199905270-00004

[12] Nishi H Hanafusa N Kondo Y Clinical outcome of thrombotic microangiopathy after living-donor liver transplantation treated with plasma exchange therapy. *Clin J Am Soc Nephrol* 2006; 1:811–819. 17699291 10.2215/CJN.01781105

[13] Darius T Monbaliu D Aerts R Rescue of a marginal liver graft by sequential treatment with molecular adsorbent recirculating system and transjugular intrahepatic portosystemic shunt: a case report. *Transplant Proc* 2009; 41:3427–3429. 19857762 10.1016/j.transproceed.2009.09.018

[14] Yagi S Iida T Hori T Optimal portal venous circulation for liver graft function after living-donor liver transplantation. *Transplantation* 2006; 81:373–378. 16477223 10.1097/01.tp.0000198122.15235.a7

[15] Feng AC Liao CY Fan HL A successful child-to-adult deceased donor liver transplantation: a case report and literature review. *Ann Transplant* 2015; 20:21–24. 25582243 10.12659/AOT.893101

[16] Akamatsu N Sugawara Y Satou S Hemodynamic changes in the hepatic circulation after the modulation of the splenic circulation in an in vivo human experimental model. *Liver Transpl* 2014; 20:116–121. 24123877 10.1002/lt.23763

[17] Neumann UP Langrehr JM Kaisers U Simultaneous splenectomy increases risk for opportunistic pneumonia in patients after liver transplantation. *Transpl Int* 2002; 15:226–232. 12012043 10.1007/s00147-002-0399-8

[18] Samimi F Irish WD Eghtesad B Role of splenectomy in human liver transplantation under modern-day immunosuppression. *Dig Dis Sci* 1998; 43:1931–1937. 9753254 10.1023/a:1018822206580 PMC2977917

[19] Tarantino G Savastano S Capone D Spleen: a new role for an old player? *World J Gastroenterol* 2011; 17:3776–3784. 21987619 10.3748/wjg.v17.i33.3776 PMC3181438

[20] Di Sabatino A Carsetti R Corazza GR Post-splenectomy and hyposplenic states. *Lancet* 2011; 378:86–97. 21474172 10.1016/S0140-6736(10)61493-6

[21] Rubin LG Schaffner W Clinical practice. Care of the asplenic patient. *N Engl J Med* 2014; 371:349–356. 25054718 10.1056/NEJMcp1314291

[22] Yasutomi M Innocenti HS DeSouza F RA. Outcome of the use of pediatric donor livers in adult recipients. *Liver Transpl* 2001; 7:38–40. 11150420 10.1053/jlts.2001.18482

[23] Emre S Soejima Y Altaca G Safety and risk of using pediatric donor livers in adult liver transplantation. *Liver Transpl* 2001; 7:41–47. 11150421 10.1053/jlts.2001.20940

[24] Zhang L Zeng L Gao X Transformation of organ donation in China. *Transpl Int* 2015; 28:410–415. 25267538 10.1111/tri.12467

[25] Foley DP Impact of donor warm ischemia time on outcomes after donation after cardiac death liver transplantation. *Liver Transpl* 2014; 20:509–511. 24677445 10.1002/lt.23859

[26] Gonwa TA Mai ML Melton LB Renal replacement therapy and orthotopic liver transplantation: the role of continuous veno-venous hemodialysis. *Transplantation* 2001; 71:1424–1428. 11391230 10.1097/00007890-200105270-00012

[27] Lopez-Andujar R Oron EM Carregnato AF T-tube or no T-tube in cadaveric orthotopic liver transplantation: the eternal dilemma: results of a prospective and randomized clinical trial. *Ann Surg* 2013; 258:21–29. 23426348 10.1097/SLA.0b013e318286e0a0

[28] Gastaca M Valdivieso A Ruiz P T-tube or no T-tube in cadaveric orthotopic liver transplantation: the type of tube really matters. *Ann Surg* 2015; 261:e172. 23799419 10.1097/SLA.0b013e31829d56c0

[29] Kotloff RM Blosser S Fulda GJ Management of the potential organ donor in the ICU: Society of Critical Care Medicine/American College of Chest Physicians/Association of Organ Procurement Organizations Consensus Statement. *Crit Care Med* 2015; 43:1291–1325. 25978154 10.1097/CCM.0000000000000958

